# Central nervous system regeneration: the roles of glial cells in the potential molecular mechanism underlying remyelination

**DOI:** 10.1186/s41232-022-00193-y

**Published:** 2022-03-02

**Authors:** Lili Quan, Akiko Uyeda, Rieko Muramatsu

**Affiliations:** Department of Molecular Pharmacology, National Institute of Neuroscience, National Center of Neurology and Psychiatry, 4-1-1 Ogawa-higashi, Kodaira, Tokyo, 187-8502 Japan

**Keywords:** Central nervous system, Glial cell, Remyelination, Systemic factors, Multiple sclerosis

## Abstract

Glial cells play crucial roles in brain homeostasis and pathogenesis of central nervous system (CNS) injuries and diseases. However, the roles of these cells and the molecular mechanisms toward regeneration in the CNS have not been fully understood, especially the capacity of them toward demyelinating diseases. Therefore, there are still very limited therapeutic strategies to restore the function of adult CNS in diseases such as multiple sclerosis (MS). Remyelination, a spontaneous regeneration process in the CNS, requires the involvement of multiple cellular and extracellular components. Promoting remyelination by therapeutic interventions is a promising novel approach to restore the CNS function. Herein, we review the role of glial cells in CNS diseases and injuries. Particularly, we discuss the roles of glia and their functional interactions and regulatory mechanisms in remyelination, as well as the current therapeutic strategies for MS.

## Introduction

**Fig. 1:**
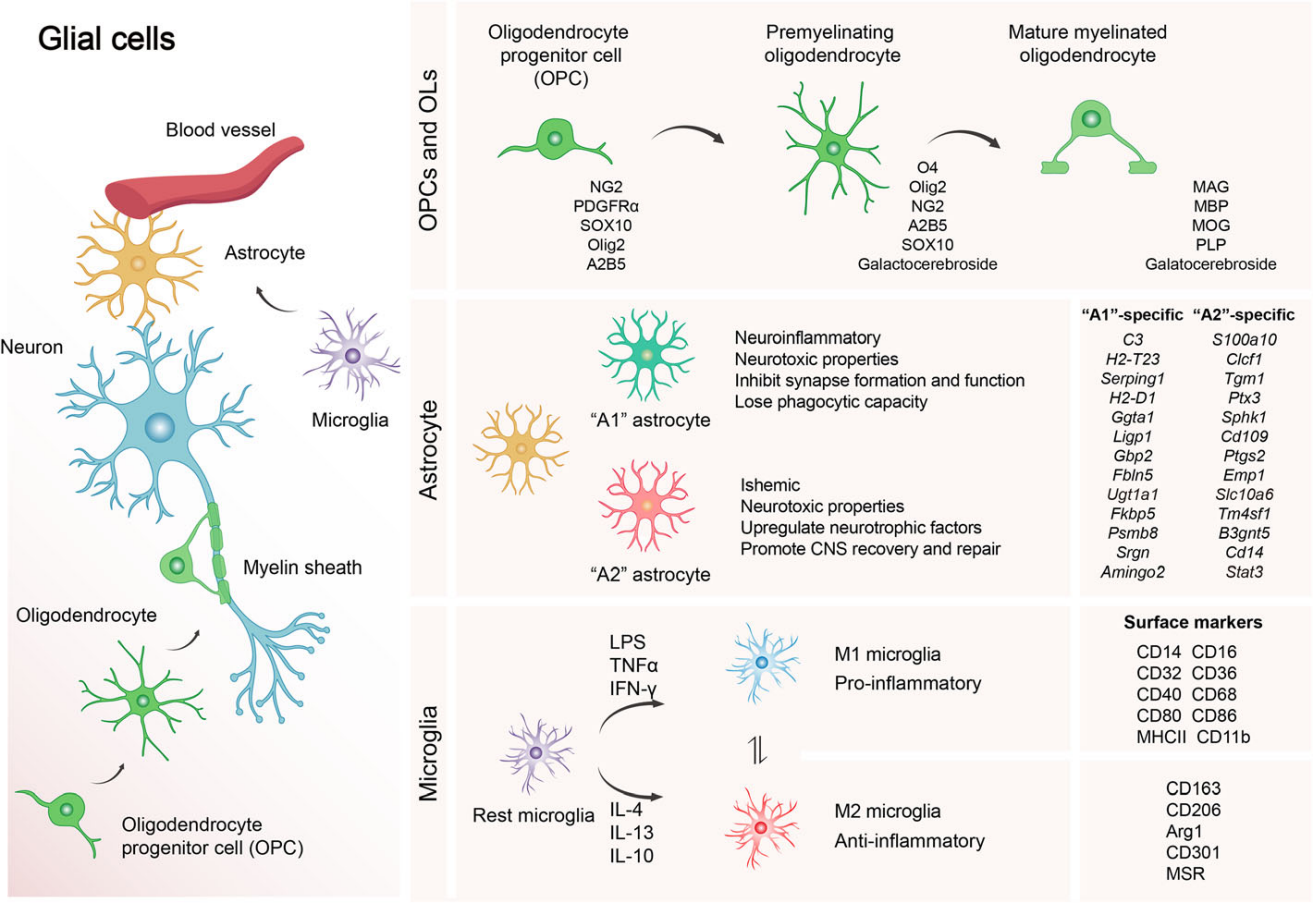
Schematic representation of glial cells in the CNS, along with their classification and representative marker genes. Oligodendrocytes, astrocytes, and microglia are the major population of glial cells in CNS. Oligodendrocytes (OLs) are myelinating cells derived from oligodendrocyte progenitor cells (OPCs). OLs and OPCs are important cellular components for remyelination, which is an important regenerative process to restore lost neurological function. OPCs express PDGFRα (platelet-derived growth factor receptor α), the transcription factor SOX10, NG2, Olig2, and A2B5. After OPCs generate OLs, the cells start to express O4, galactocerebroside. Finally, mature myelinating OLs express myelin-associated glycoprotein (MAG), myelin basic protein (MBP), proteolipid protein (PLP), and myelin oligodendrocyte glycoprotein (MOG). Astrocytes are the most abundant cells in the mammalian CNS with the function of forming and maintaining blood-brain barrier (BBB), regulating regional blood flow, proving trophic, antioxidant and metabolic support to neurons, neurotransmitter recycling, immune signaling, and regulating neuronal synaptogenesis and synaptic transmission. Reactive astrocytes can be classified into two subtypes, termed as “A1” (neurotoxic)/ “A2” (neuroprotective) astrocytes, with the specific marker genes identified from the previous transcriptome analysis [49]. Microglia can be classified into two states: M1 (classic) or M2 (alternative), which are responsible for tissue injury and repair, respectively

Adult central nervous system (CNS) has a limited regenerative capacity following injury or disease. Glial cells (astrocytes, oligodendrocytes, and microglia) are the major glial populations of the CNS and are crucial for brain development and homeostasis as well as the pathological process (Fig. 1). Remyelination is a regenerative process in the CNS, which occurs spontaneously and counters demyelinating diseases to restore lost neurological deficits and axonal function [1, 2]. Successful remyelination depends on the involvement of multiple cell types around the lesion site, and glial cells are one of the most important cell groups in this process [3]. With aging, diminished activity of progenitor cells such as oligodendrocyte precursor cells (OPCs) within the nervous system means that reversal of the damage caused by CNS diseases and injuries becomes increasingly impossible [2]. Currently, there are no effective therapies in regard to treating the demyelinating diseases. Thus, illustrating the underlying molecular mechanism of remyelination is beneficial to the development of therapeutic strategies for CNS injuries and diseases. In this review, we discussed the properties of glia and their functions in CNS remyelination. Notably, we emphasized the role of systemic factors regulating the processes of OPCs in remyelination and glia-glia interaction in this process, providing a novel direction to combat the challenges of demyelination caused by injury, diseases, and aging of the brain. Furthermore, we summarized the therapeutic strategies that are currently being used in demyelinating disease-multiple sclerosis (MS), paving the way for the development of therapeutics for CNS remyelination.

## Main text

### Oligodendrocyte progenitor cell and oligodendrocyte

Oligodendrocyte progenitor cells, also known as oligodendrocyte precursor cells (OPCs), which express proteoglycan neuron-glial antigen 2 (NG2), are the main dynamic, proliferative cells within the adult CNS [4]. Mature oligodendrocytes (OLs) are differentiated from OPCs during the embryogenesis and early stages of postnatal life [5]. Moreover, in adult, there is still an undifferentiated population of OPCs maintained in the CNS, which hold the capacity to continue into mature OLs through activation, proliferation, migration, and differentiation for the adaptive myelin sheath formation [6]. Multiple transcriptional factors and growth factors play essential roles in oligodendrocyte differentiation and maturation [7–15]. For example, in response to a demyelinating insult, OPCs are recruited to the demyelination areas and activated by the upregulation of transcription factors, such as Olig1, Olig2, Nkx2.2 [7], Sox10 [10], and Sox2 [11]. Subsequently, OPCs proliferate under the modulation of growth factors such as fibroblast growth factor (FGF) and platelet derived growth factor (PDGF) from the CNS microenvironment [3], and finally differentiate into mature OLs to form the myelin sheaths that wrap the exposed demyelinated axons under the regulation of factors including myelin regulatory factor (Myrf) [12], TCF7L2 [13, 14], and Sox17 [15]. According to the protein expression pattern of the development of oligodendrocyte, multiple markers have been developed to recognize the cell at different stages [16] (Fig. 1). However, the potential of OPCs for differentiation is different between brain regions and age, which is correlated with electrophysiological changes and molecular or cellular environments [17–19].

The progression of OPCs is increasingly implicated in neurodegenerative diseases such as MS, as OLs not only myelinate, but also provide metabolic support for axons [20]. Traditionally, OPCs, rather than pre-existing mature oligodendrocytes, have been identified as the cells most responsible for remyelination [21]. Surprisingly, recent evidences suggest that adult oligodendrocyte also can participate in remyelination [16]. Through the light and electron microscopy, they found that oligodendrocytes are connected to remyelinated and mature myelin sheaths. In vitamin B_12_-deficienct nonhuman primates demyelination model, it shows that survived mature oligodendrocyte is capable to extend and connect demyelinated axons, demonstrating that partially injured mature oligodendrocytes also hold the ability to participate in myelin repair of demyelinating diseases [22]. More recently, study using single-nucleus RNA sequencing revealed seven clusters of OLs and additional OPCs clusters with specific or enriched RNA markers for each subclusters in the white matter of MS patient brain, suggesting the heterogeneity of oligodendrocyte may relate to different functional states of oligodendrocytes in MS progression [20]. Additionally, Yeung, M.S.Y [23] uncovers oligodendrocyte generation dynamics in MS using carbon-dated strategy to measure the ^14^C level in genomic DNA of oligodendrocytes from different stages. Enrichment-based approaches of MS genome-wide association studies SNPs nominate the oligodendrocyte intrinsic contribution in MS pathogenesis via the disruption of RNA polymerase II release [24]. Together, those results indicate that OLs and OPCs are key players in MS. Beyond MS, dysfunction of oligodendrocyte is also found in schizophrenia and bipolar disorder [25]. OPCs senescence induced by β-amyloid plays a role in neuroinflammation and cognitive deficits in Alzheimer’s disease (AD) [26].

#### Regulation of systemic factors of remyelination

**Fig. 2:**
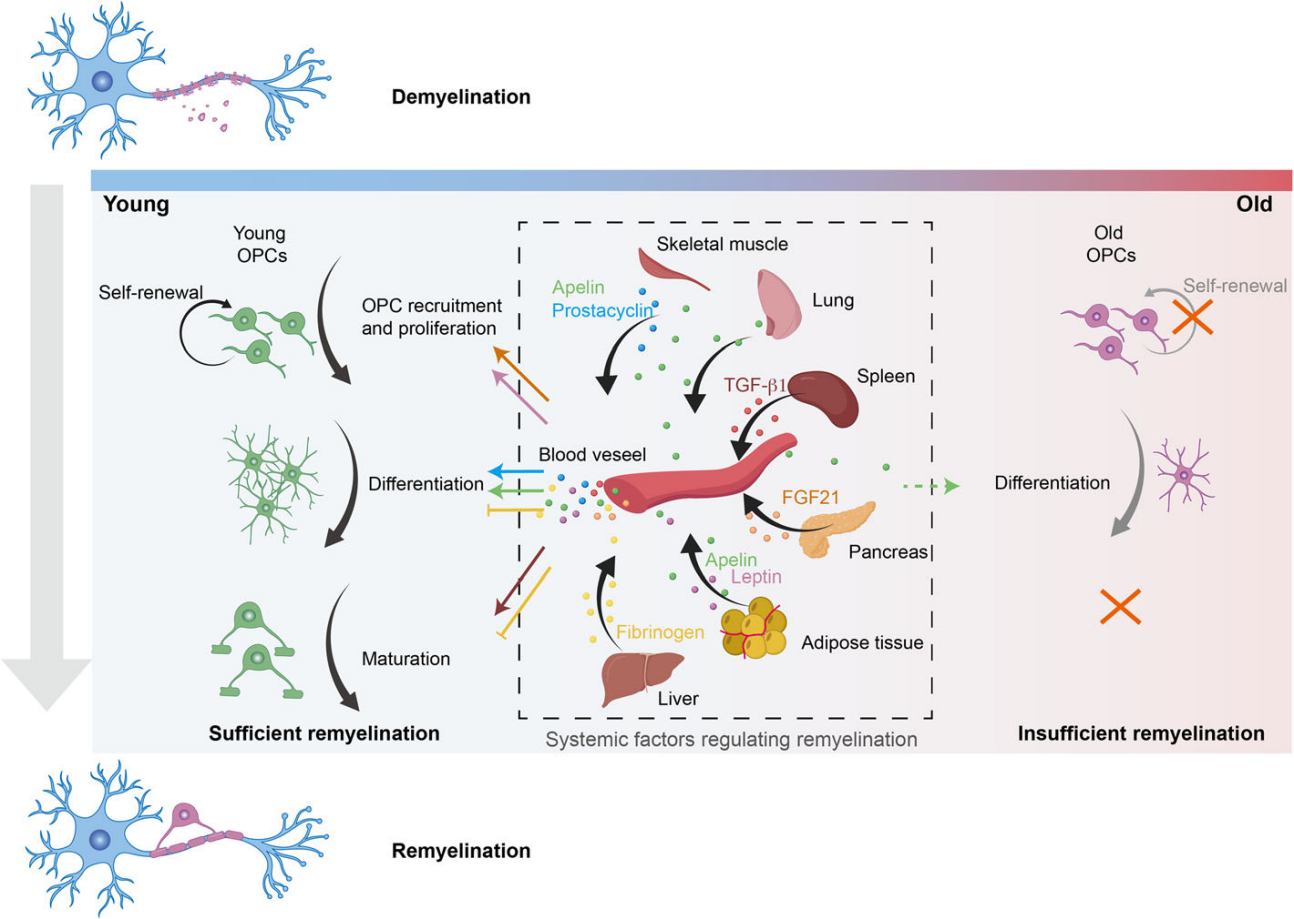
Molecular mechanism of remyelination in CNS. In response to the CNS injury to myelinate axons, remyelination is initiated with oligodendrocyte progenitor cells (OPCs) recruitment and activation to the lesion site. Then followed by OPCs proliferation, differentiation, and maturation to mature oligodendrocyte for myelin sheath formation. CNS injuries often along with the disruption of blood-brain barrier (BBB) in the lesion site. Systemic factors leaked from BBB contribute to the remyelination through promoting OPCs proliferation, differentiation, and maturation. Pancreas-derived FGF21 and adipose tissue-derived leptin promotes OPCs proliferation; skeletal muscle-derived prostacyclin, and lung, muscle, adipose tissue-derived apelin are responsible for the oligodendrocyte differentiation; spleen-derived transforming growth factor-β1 in serum induces the oligodendrocyte maturation. In contrast, fibrinogen disrupts OPCs differentiation and myelination upon BBB disruption. Therapeutic depletion of fibrinogen will be benefit for remyelination. OPCs, similar to other adult stem cell, undergo a functional decline with ageing. The ability of OPCs to self-renew and to differentiate is diminished. Also, the efficiency of oligodendrocyte differentiation is declined with age. In particular, the expression of apelin receptor (APJ) in oligodendrocytes is decreased and result in an insufficient remyelination. Alternatively, activation of APJ promotes the remyelination both in aged mice and toxin-induced EAE. Therefore, activation of apelin/APJ signaling restores the function of oligodendrocyte for differentiation and thereby enhance its capacity for remyelination

From the previous published results using the in vitro oligodendrocytes culture or in vivo zebrafish and mouse model, showing that multiple extrinsic factors, as well as systemic factors are responsible for remyelination. Some of those factors or receptors have been regarded as potential targets for the development of drugs to treat demyelinating-related diseases, such as MS [27]. Currently, illustration of the pathophysiological function of circulating factors from vascular disruption has been emerged to be novel strategies toward remyelination for CNS regeneration (Fig. 2). Based on the previous studies, we described that multiple circulating factors released from the bio-system exhibit essential roles in OPC development, for example the fibrinogen [28], fibroblast growth factor 21 (FGF21) [29], transforming growth factor-β1 (TGF-β1) [30], and apelin [31].

##### Fibrinogen

Fibrinogen is a blood coagulation protein synthesized in liver [32]. Upon blood-brain barrier (BBB) disruption, fibrinogen leaks into the brain, and, by activation of the bone morphogenetic protein (BMP) pathway to inhibit oligodendrocyte differentiation and remyelination. Exposing OPCs to fibrinogen in vitro, the differentiation of OPCs into OLs is inhibited as shown by the decreased MBP expression. Through the OPC/dorsal root ganglion myelinating and fibrinogen-coated nanofiber myelinating culture system, they found that fibrinogen significantly suppresses the MBP^+^ OLs and myelin sheath formation, suggesting that fibrinogen inhibits the oligodendrocyte maturation. In order to understand the mechanism about how fibrinogen affects OPC differentiation, the authors used whole-genome microarray and gene ontology analysis and found that BMP responsive genes (*Id1*, *Id2*, *Nog*, *Hes1*, *Hey1*, and *Lef1*) were upregulated upon fibrinogen treatment of OPCs. With the treatment of BMP type I receptor ACVR1 inhibitor, DMH1 or CRISPR/Cas9 *ACVR1* knockout, the inhibitory effect of fibrinogen on OPCs was significantly blocked, suggesting that fibrinogen activates the BMP signaling via ACVR1 to show its inhibitory effect on OPCs differentiation and myelin production. Depletion of fibrinogen in lysophosphatidylcholine (LPC)-induced demyelination mouse model, the BMP signaling is inhibited and the remyelination is enhanced [28]. Thus, fibrinogen is a potential therapeutic target for promoting the remyelination and repair of demyelinating-related diseases. Interestingly, they found that OPCs exposed to fibrinogen underwent a development of astrocytes both in vitro and in vivo. Accumulating evidence suggests that blood-derived fibrinogen triggers astrocyte scar formation [33] and is involved in neural stem cell differentiation into astrocyte within the subventricular zone niche following the brain injury [34]. It is possible that the blood leakage factors may perform different but cooperative functions in multiple cellular types to achieve its function for CNS regeneration.

##### FGF21

FGF21, which is predominantly expressed in pancreas, mediates OPC proliferation by interaction with β-klotho which is an important coreceptor of FGF21 [31]. In vivo, when injection of LPC to the spinal cord to construct the demyelination with vascular barrier disruption, the FGF21 concentration was also increased as the same time course of OPC proliferation in the injury lesion. Knockout of FGF21 showed a decreased spontaneous remyelination and functional recovery following LPC injection. When silencing the expression of FGF21 in pancreas, the serum-mediated OPC proliferation was significantly inhibited, indicating the critical role of pancreas-derived FGF21 in OPCs proliferation and CNS remyelination [29].

##### TGF-β1

TGF-β1, which shows appreciable expression in the spleen, promotes oligodendrocyte maturation for the achievement of remyelination and functional recovery of myelin oligodendrocyte glycoprotein (MOG)_35-55_ induced EAE model. With the treatment of adult mouse serum, the oligodendrocyte maturation is promoted dramatically via TGF-β type I receptor, as assessed via the determination of the MBP-positive area. Similarly, the enhancement of oligodendrocyte maturation induced by TGF-β1 was also observed in human samples assessed by the mRNA level of myelin-associated proteins, MBP, MAG, and PLP, using quantitative RT-PCR in vitro. Administration of TGF-β1 to demyelinating animal models promotes the remyelination and functional recovery. These results suggest that circulating factors from the disruption of vascular barrier play critical roles in demyelinating-diseases [30].

##### Apelin

Apelin/APJ improves oligodendrocyte differentiation during ageing and age-related pathologies to promote remyelination. By comparing the RNA expression profile data from developing and aged mice focusing on the genes associated with the differentiation markers, *Aplnr* appears to be a potential biomarker of oligodendrocyte differentiation and responsible for the promotion of rejuvenation of aged-related dysfunction of remyelination. The expression of APJ in myelin-forming cells decreases with age, and is correlated with a decrease in myelin-associated genes. The expression of *Apln* is detected in multiple tissues, including the lung, adipose tissue, and skeletal muscle, all of which also exhibit reduced *Apln* expression in aged animals. APJ agonist ML233 treatment in aged mice with LPC-induced demyelination resulted in the recovery of neurological function and promotion of remyelination compared to that in the control mice. In addition, the oligodendrocyte maturation was also confirmed by upregulated-expression of myelin-associated proteins (Mbp, Plp, and Mog) in human OPC with the stimulation of ML233. Thus, it is promising that the dysfunction of apelin/APJ signaling is associated with the failure of ageing-related demyelination, indicating a novel critical role of circulating factors in age-related CNS remyelination [31].

##### Other factors

Besides the above discussed circulating factors, endocrine hormones secreted from muscle cells promote OPCs proliferation and myelination [35]; heart-derived factors hold the ability to promote OPCs proliferation through the phosphorylation of phosphatidylinositol 3-kinase and extracellular signal-regulated kinase [36]; the adipose tissue abundant leptin is also responsible for the promotion of OPC proliferation in toxin-induced demyelination model and in vitro [37]. Taken together, it is no doubt that systemic factors are essential mediators of brain homeostasis, ageing, and neurodegeneration [38], in particular, circulating factors and its corresponding receptors are potential targets for remyelination and for the development of drugs for CNS diseases.

### Astrocyte

Astrocytes, the most abundant glial cells, play a critical role in both physiology and pathology processes of CNS. It is becoming clear that astrocytes hold a wide range of functions: associating with endothelial cells, neurons, and pericytes to form and maintain BBB; regulating regional blood flow; proving trophic, antioxidant and metabolic support to neurons, neurotransmitter recycling, immune signaling; and regulating neuronal synaptogenesis and synaptic transmission [39–45]. In response to CNS injury, astrocytes will turn into reactive astrocytes with a notable feature of the upregulation of glial fibrillary acidic protein (GFAP) at both of the mRNA and protein levels. Thus, GFAP has been widely regarded as a marker for reactive astrocytes. However, only focusing on GFAP and morphology are not sufficient to evaluate astrocytes as reactive, because the degree of GFAP expression in reactive astrocytes is modulated due to the regional difference, the proximity to the injury site, and the type or severity of injuries [46]. Therefore, combination of molecular markers (GFAP with other astrocyte markers such as aldehydedehydrogenase-1 L1, S100B, glutamine synthetase, aldolase-C) and functional readouts is recommended to evaluate the astrocyte phenotypes [47].

Reactive astrocytes can classified into two subtypes; “A1” (neurotoxic) and “A2” (neuroprotective) astrocytes, based on transcriptomic and epigenetic studies that profiled reactive astrocytes from lipopolysaccharide-induced neuroinflammation or middle cerebral artery occlusion-induced ischemic stroke mouse model separately [48] (Fig. 1) . Compared to normal astrocytic functions, “A1” astrocytes lose their phagocytic capacity and their capacity to clear myelin debris in vivo after CNS injury [49]. Thus, the inhibition of “A1” astrocyte formation after acute CNS injury is presumed to prevent the death of axotomized neurons [50]. Whereas, “A2” astrocytes upregulate multiple neurotrophic factors and play a protective role in promoting CNS recovery and repair [49]. However, the binary classification of reactive astrocytes (A1/A2) has been challenged by single-cell and single-nucleus RNA-seq analyses, and spatial transcriptomics in the tissue samples from various CNS diseases such as AD [51], Huntington’s disease [52, 53], and amyotrophic lateral sclerosis [54]. Thus, the previously profiled marker genes classified as either “A1/neurotoxic” or “A2/neuroprotective” is speculative and needs to be further investigated, and more comprehensive single-cell genomic analyses and representative models are needed to accelerate our understanding of astrocyte subtype-specific profiles. Ongoing single-cell genomic studies, spatial transcriptomics, and conditional knockdown or knockout experimental models [55, 56] have enabled the investigation of the morphology and physiology of different distributions of astrocyte populations under normal or pathological conditions. Thus, in the future, it may be possible to distinguish astrocyte subtypes in a more comprehensive manner.

#### Roles of astrocytes in remyelination

**Fig. 3:**
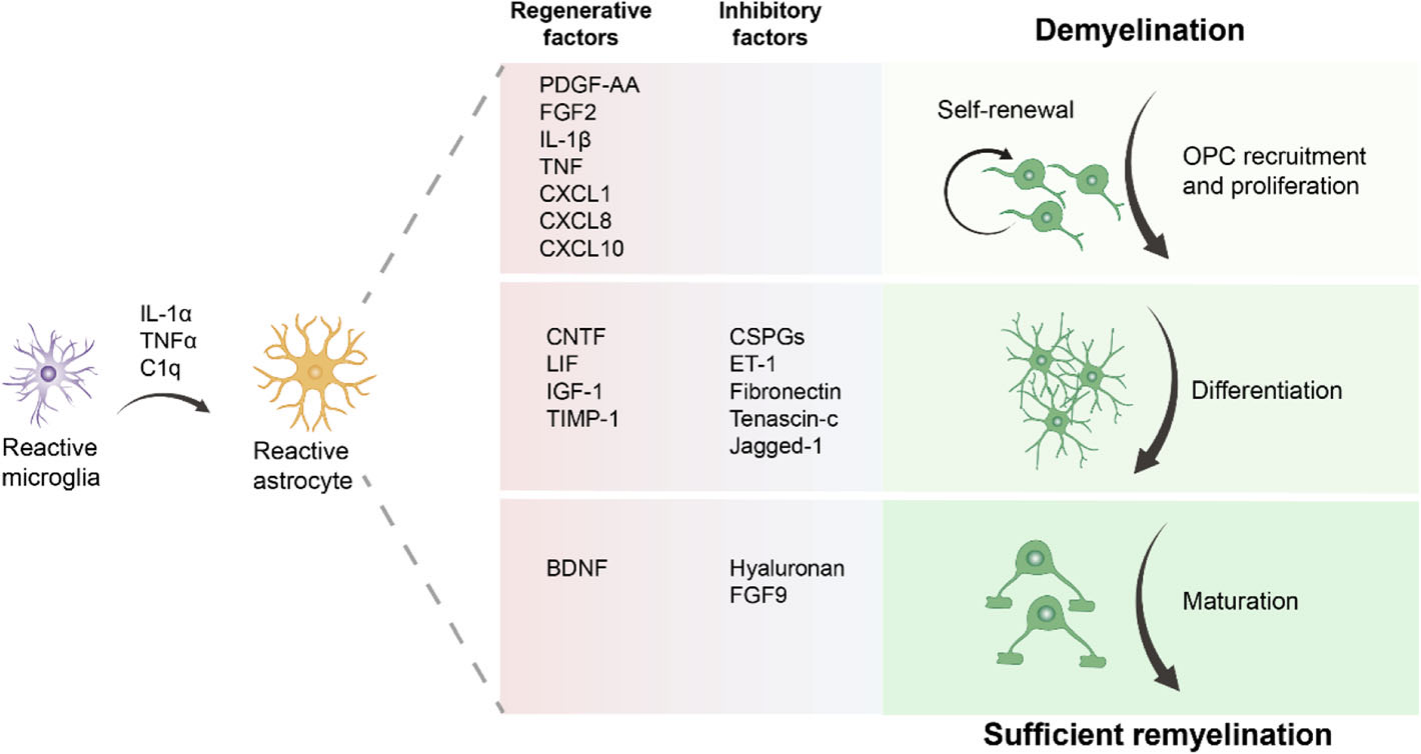
Roles of astrocytes in remyelination. Reactive astrocytes are induced by cytokines (interleukin [IL]-1α, TNF, and C1q) secreted by activated microglia in response to demyelinating insults. Astrocytes regulate the proliferation, differentiation, and maturation of OPCs by secreting regenerative or inhibitory factors that modulate remyelination. Astrocytes promote OPC proliferation by producing growth factors such as platelet-derived growth factor AA (PDGF-AA), fibroblast growth factor 2 (FGF2), cytokines IL-1β and tumor necrosis factor (TNF), and chemokine C-X-C motif chemokine ligand 1, 8, and 10 (CXCL1, CXCL8, CXCL10). Astrocytes increase OPC differentiation by secreting ciliary neurotrophic factor (CNTF), leukemia inhibitory factor (LIF), insulin-like growth factor-1 (IGF-1), and tissue inhibitor of metalloproteinases-1 (TIMP-1). In contrast, astrocytes also produce inhibitory molecules, such as chondroitin sulfate proteoglycans (CSPGs), endothelin-1 (ET-1), fibronectin, tenascin-c, and jagged-1. Furthermore, astrocytes produce brain-derived neurotrophic factor (BDNF) to promote hyaluronan and fibroblast growth factor 9 (FGF9) to suppress the maturation of OLs

The details of the function of astrocytes in remyelination toward OPCs or other cell types such as Schwann cells have been widely discussed elsewhere [57]. In brief, reactive astrocytes were induced by activated microglia-secreted cytokines (interleukin-1α [IL-1α], tumor necrosis factor [TNF], and C1q) in response to demyelinating insults. Astrocytes exhibit a dual function during remyelination by secreting either regenerative or inhibitory factors, which modulate the proliferation, differentiation, and maturation of OPCs (Fig. 3). Astrocytes promote OPC proliferation through growth factors platelet-derived growth factor AA (PDGF-AA), FGF2, cytokines interleukin-1β (IL-1β), TNF, and chemokine C-X-C motif chemokine ligand 1, 8, and 10 (CXCL1, CXCL8, CXCL10) [58–62]. Astrocytes also promote OPC differentiation by secreting ciliary neurotrophic factor (CNTF) [63], leukemia inhibitory factor (LIF) [64], insulin-like growth factor-1 (IGF-1) [65], and tissue inhibitor of metalloproteinases-1 (TIMP-1) [66]. Moreover, astrocytes produce inhibitory molecules, such as chondroitin sulfate proteoglycans (CSPGs) [67], endothelin-1 (ET-1) [68], fibronectin [69], tenascin-c [70], and jagged-1 [71] which have an inhibitory role in remyelination, as well as brain-derived neurotrophic factor (BDNF), hyaluronan, and fibroblast growth factor 9 (FGF9), to facilitate or suppress the maturation of OLs [72–74] (Fig.3). In addition to the secretion of regulatory factors to regulate remyelination as described above, astrocytes also play a role in mediating copper transport [75] and recruiting microglia into demyelinating lesions to promote their phagocytic function [76]. Recent evidence suggests that astrocyte-mediated copper transport promotes demyelination and tissue injury [75]. This study further finds that astrocyte-dependent copper is distributed in the white matter of human and mouse models of MS. It is possible that reducing the distribution of copper may sufficiently promote remyelination and tissue repair [75]. Furthermore, astrocytes may also support remyelination by controlling cholesterol synthesis and transfer via apolipoprotein E [77, 78].

### Microglia

Microglia can be defined as both glia and resident macrophage-like immune cells of CNS, which play a critical role in CNS development, homeostasis, and in response of brain injury. In the physiological conditions, microglia interact with the surrounding neurons, astrocytes, and oligodendrocytes to show its homeostatic function through cell-to-cell communication and synapse formation [79]. In the pathological conditions, microglia have the capacity to change the morphology rapidly and response to the stimuli adoptively in numerous CNS disorders. In response to acute CNS injury, the activation of microglial is induced in both mammals and zebrafish [80]. Growing evidence indicates that microglial necroptosis and repopulation drive CNS remyelination [81]. Traditionally, microglia can be classified into two states: M1 (classic) and M2 (alternative) state (Fig. 1). M1 is a pro-inflammatory state with the upregulation of MHC class II and the release of pro-inflammatory cytokines, IL-1β, IL-6, IL15, TNF-α, and IFN-γ. M2 is an anti-inflammatory state with the production of trophic factors, like tumor growth factor-β (TGF-β), IL-10, growth factor IGF-1, and BDNF [82]. In the classical conversion of microglia to M1 microglia, the NF-κB and iNOS pathways are upregulated to defend the tissue and facilitate the destruction of invading pathogens in response to injury and infection. However, M1 microglia also induce neurotoxicity by producing multiple pro-inflammatory cytokines and inducing acute inflammation. The activation of M1 microglia induces the expression of iNOS, and the depletion of iNOS expression sufficiently protects AD mice from cerebral plaque formation and premature mortality, and increases the AD protective response [83]. After classical activation, anti-inflammatory and repair processes are rapidly initiated with the activation of the M2 microglia, which have the potential to suppress pro-inflammatory immune responses and promote damage repair [84]. Recent studies have shown that transplantation of M2-deviated microglia rather than M1 microglia efficiently accelerates the motor function recovery in SCI mouse models [85]. Moreover, M2-derived activin-A drives the differentiation of oligodendrocytes, indicating that M2 cell polarization is crucial for successful remyelination [86].

#### Roles of the microglia in remyelination

**Fig. 4:**
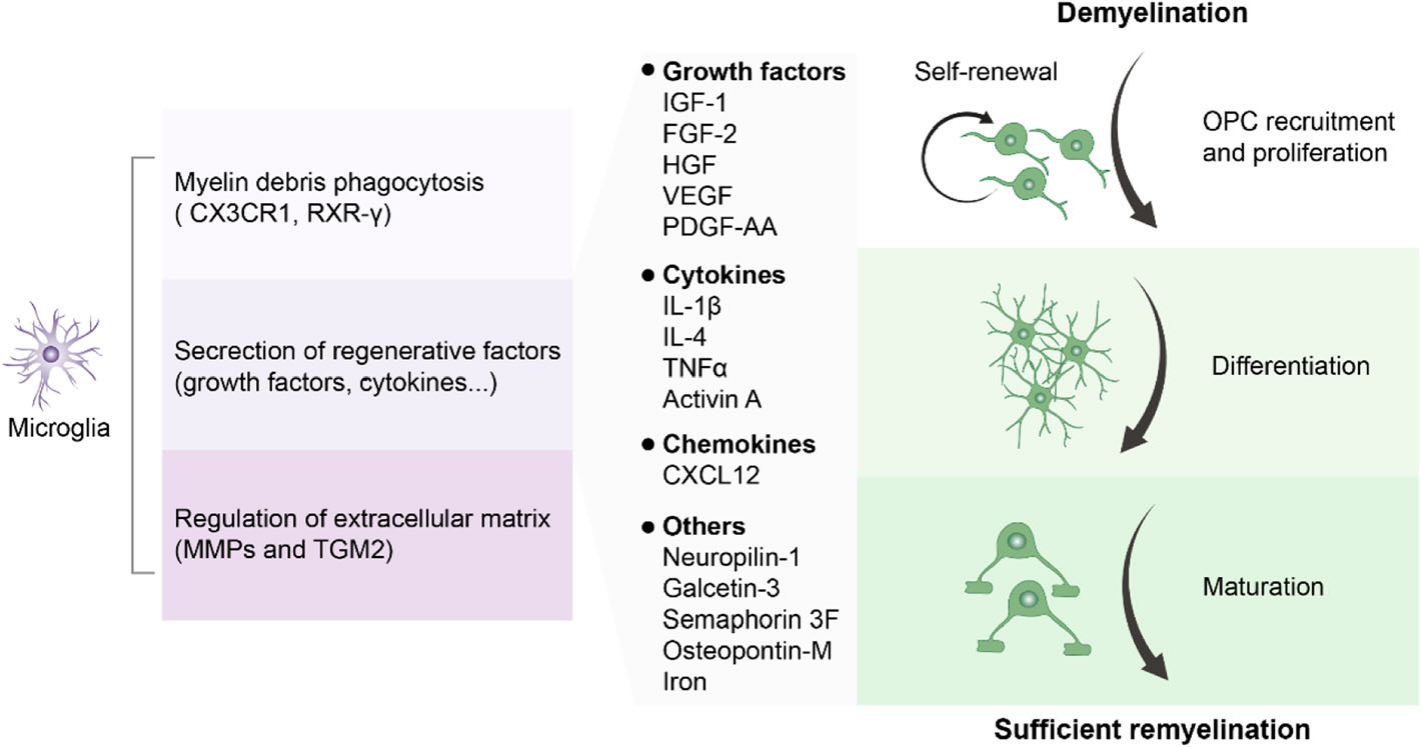
Roles of microglia in remyelination. Microglia promote remyelination to facilitate OPC proliferation and differentiation for myelin formation by the following three mechanisms: (1) myelin debris phagocytosis (via CX3CR1 and RXR-γ receptors). (2) Secretion of regenerative factors such as growth factors, insulin-like growth factor 1 (IGF-1), fibroblast growth factor-2 (FGF-2), hepatocyte growth factor (HGF), vascular endothelial growth factor (VEGF), platelet-derived growth factor AA (PDGF-AA); cytokines, interleukins-1β and 4 (IL-1β, IL-4), tumor necrosis factor α (TNFα), activin A; chemokine, C-X-C motif chemokine ligand 12 (CXCL12); and others, including neuropilin-1, galectin-3, semaphorin 3F, osteopontin-M, and iron. (3) Modulation of the extracellular matrix by secretion of matrix metalloproteinases (MMPs) and TGM2

Microglia promote remyelination by facilitating the proliferation and differentiation of OPCs in three manners: (1) phagocytosis of myelin debris, (2) secretion of regenerative factors, and (3) regulation of the extracellular matrix [87] (Fig. 4). The phagocytosis of myelin debris is regulated by the CX3CR1 and RXR-γ receptors expressed in the microglia [88, 89]. CX3CR1 knockout mice exhibit diminished myelin debris clearance after cuprizone-induced demyelination [88]. RXR-γ, whose expression decreases with age, coincides with impaired debris clearance after demyelination [89]. The phagocytosis of myelin debris can be reduced by antagonism or knockout [90]. Recent studies have further highlighted the role of microglia in myelin debris phagocytosis. The depletion of microglia following treatment with PLX5622 (an inhibitor of colony stimulating factor 1 receptor, CSF1R) in mice in the later stage of delayed viral clearance induced by coronavirus infection, microglia-depleted mice fail to recover clinically from hindlimb paralysis, or to resolve demyelinating lesions [91]. Microglia also regulate OPC proliferation and OL differentiation by secreting various pro-regenerative factors, including growth factors, insulin-like growth factor 1 (IGF1), FGF2, hepatocyte growth-factor (HGF), vascular endothelial growth factor (VEGF), PDGF-AA; cytokines, IL-1β, IL-4, TNF, and activin A (a member of the TGF-β superfamily); chemokine, C-X-C motif chemokine ligand 12 (CXCL12), and others including neuropilin-1, galectin-3, semaphorin 3F, osteopontin-M, and iron [90]. Additionally, the function of IL-4 in microglia has been shown to enhance oligodendrogenesis in EAE mice and promote oligodendrocyte differentiation during remyelination [92]. Recent studies have also revealed the mechanism underlying the transition of pro-inflammatory to pro-regenerative microglia during remyelination [81] (Fig. 4). RNA sequencing using isolated microglia from myelin toxin LPC-induced focal demyelinated lesions in the corpus callosum of young adult mice illustrated that microglial repopulation is positively regulated by type-1 IFN signaling during white matter remyelination, suggesting that targeting inflammatory microglia is a potential strategy for CNS white matter inflammation [81].

### MS

MS, a widely occurring demyelinating disease of the CNS, is characterized by continuous inflammation, demyelination, and axonal loss [93]. In clinical practice, immunotherapies have been widely developed for the treatment of MS, including ocrelizumab, an anti-CD20 monoclonal antibody, which was approved in 2017 by the Food and Drug Administration for the treatment of primary progressive MS [94]. However, the precaution of disability in MS patients from axonal and neuronal injury and loss has not been achieved due to incomplete or inadequate remyelination and is therapeutically challenging in clinical settings [95]. Therefore, promoting remyelination may have significant implications for improving disability in patients with MS.

Currently, multiple in vivo models have been developed to screen for potential therapeutic targets of remyelination [27]. EAE is the most common use animal model of MS, which can be induced by myelin components, such as MOG, MBP, or PLP. In clinical, MS is categorized into four courses: (1) relapsing-remitting MS (RRMS), the most common form of MS which affect around 85% of MS patients; (2) secondary progressive MS (SPMS), developed followed by RRMS with time; (3) primary progressive MS (PPMS), around 15% patients with gradual progressive neurologic deterioration at the beginning of detectable clinical signs; and (4) progressive-relapsing MS (PRMS), the least common form of MS [96–98]. MS as a severe neurological disease leads to a wide range of symptoms to the patients. Hence, multiple drugs have been developed with the aim of promoting remyelination in MS patients in clinical settings, as described in Table 1. Although several pharmacologic manipulations of MS have been developed, a better understanding of neuroprotective effect of the available drugs and the development of drugs targeting new potential molecular targets will be needed to further contribute to the management of MS patients.

**Table 1:** Drugs for MS patients targeting remyelination in clinical trials

Drug	Drug type	Details	Subtype	ClinicalTrails.gov identifier
GNbAc1	Monoclonal antibody	Assess the efficacy of HERV-W Env antagonist in MS	RRMS	NCT02782858
GSK239512	Histamine H, receptor antagonist	Evaluate whether it can remyelinate lesions in patients	RRMS	NCT01772199
Opicinumab (BIIB033)	Anti-lingo-1 monoclonal antibody	Efficacy, safety and dose tolerability for CNS remyelination	RRMS	NCT01864148
Domperidone	Dopamine D2 antagonist	Study myelin repair in patients with enhancing lesion by MRI	RRMS	NCT02493049
Clemastine Fumarate	Antihistaminic	Assessment of its tolerability as a remyelinating agent	RRMS	NCT02040298
Vaginal estriol	Estrogen	Evaluate the efficiency and remyelination role in female patients with urogenital symptoms	RRMS	NCT03774407
Liothyronine	Thyroid hormone	Safety and dose finding study of remyelination	RRMS, SPMS, PPMS	NCT02760056
Olesoxime	Cholesterol-like neuroprotectant	Measurement of remyelination by MRI	RRMS	NCT01808885
Natalizumab	Anti-integrin monoclonal antibody	Evaluate the remyelination capacity	RRMS	NCT00937677
Quetiapine fumarate	Atypical Antipsychotic	Safety and tolerability of this remyelinating agent in patients	RRMS, RPMS	NCT02087631
Alemtuzumab	Anti-CD52 monoclonal antibody	MRI measurement of remyelination	RRMS	NCT01395316
Adrenocorticotropic hormone (ACTH)	Hormone	Evaluate the effect of ACTH for treating demyelination in patients	RRMS, SPMS	NCT00854750
Bazedoxifene acetate	Selective estrogen receptor modulator	Test the efficacy of the drug as a remyelinating agent	RRMS	NCT04002934

Data listed in the table are collected from ClinicalTrials.gov by searching the terms “remyelination” within the condition of “multiple sclerosis.” *RRMS* relapsing remitting multiple sclerosis, *SPMS* secondary progressive multiple sclerosis, *PPMS* primary progressive MS, *CNS* central nervous system, *HERV* human endogenous retrovirus, *Env* envelope protein, *MRI* magnetic resonance imaging

## Conclusion

The long-lasting goal in regeneration toward CNS injury or diseases is to restore the lost function of nerve. To achieve this purpose, numerous cellular and molecular components are involved in the process. One approach is to replenish lost neural cells through the activation of neural stem cells (NSCs) and cell transplantation. The other approach is to manipulate the potential therapeutic target molecules of axon regeneration to achieve the functional recovery of CNS [99]. In the past decades, cell transplantation of stem cells, including ES and iPS cells appears to be an efficient therapeutic strategy for CNS injuries like SCI [100]. However, the therapeutic approach of cell transplantation contains several controversies, such as the unlimited NSCs proliferation that may lead to the tumor formation and the accuracy of cell differentiation for specific therapeutic effect [101]. Therefore, combination of cell transplantation and the knowledge of molecular mechanisms regulating cell development would be able to utilize cell transplantation therapy. Additionally, transplanted cells are also required to rebuild the neuronal network around the lesion; it is expected that the research underlying the molecular mechanisms of CNS regeneration will contribute to cell transplantation approach for treating CNS diseases. Nevertheless, successful regeneration and function restoration of CNS remain a great challenge. Although multiple potential targets have been investigated in recent studies, there is still a long journey for the development of efficient drugs for functional recovery of CNS. For this perspective, continued studies are needed to elucidate the detailed mechanism of CNS remyelination and the development of sufficient therapies for CNS injuries and diseases.

## Data Availability

Not applicable.

## Abbreviations

CNS: Central nervous system
AD: Alzheimer’s disease
MS: Multiple sclerosis
OPCs: Oligodendrocyte precursor cells
NG2: Neuron-glial antigen 2
OLs: Oligodendrocytes
PDGF: Platelet-derived growth factor
Myrf: Myelin regulatory factor
PDGFRα: Platelet-derived growth factor receptor α
MBP: Myelin basic protein
PLP: Proteolipid protein
MAG: Myelin-associated glycoprotein
MOG: Myelin oligodendrocyte glycoprotein
BBB: Blood-brain barrier
GFAP: Glial fibrillary acidic protein
TGF-β: Tumor growth factor-β
BDNF: Brain-derived neurotrophic factor
LPC: Lysophosphatidylcholines
EAE: Experimental autoimmune encephalomyelitis
VEGF: Vascular endothelial growth factor
FGF: Fibroblast growth factor
IGF: Insulin-like growth factor 1
HGF: Hepatocyte growth factor
MMPs: Matrix metalloproteinases
LIF: Leukemia inhibitory factor
TIMP-1: Tissue inhibitor of metalloproteinases 1
CSPGs: Chondroitin sulphate proteoglycans
ET-1: Endothelin-1
IL: Interleukin
TNF: Tumor necrosis factor
CXCL: C-X-C motif chemokine ligand
DDAH1: Dimethylarginine dimethylaminohydrolase 1
APJ: Apelin receptor
BMP: Bone morphogenetic protein
SCI: Spinal cord injury
RRMS: Relapsing-remitting MS
SPMS: Secondary progressive MS
PPMS: Primary progressive MS
PRMS: Progressive-relapsing MS
NSCs: Neural stem cells
